# Dynamic Variations in Multiple Bioactive Constituents under Salt Stress Provide Insight into Quality Formation of Licorice

**DOI:** 10.3390/molecules24203670

**Published:** 2019-10-11

**Authors:** Chengcheng Wang, Lihong Chen, Zhichen Cai, Cuihua Chen, Zixiu Liu, Xunhong Liu, Lisi Zou, Jiali Chen, Mengxia Tan, Lifang Wei, Yuqi Mei

**Affiliations:** 1 College of Pharmacy, Nanjing University of Chinese Medicine, Nanjing 210023, China; ccw199192@163.com (C.W.); clh_helen@163.com (L.C.); caizhichen2008@126.com (Z.C.); cuihuachen2013@163.com (C.C.); liuzixiu3221@126.com (Z.L.); zlstcm@126.com (L.Z.); 18994986833@163.com (J.C.); 18816250751@163.com (M.T.); weilifang1995@yeah.net (L.W.); 18260028173@163.com (Y.M.); 2 Collaborative Innovation Center of Chinese Medicinal Resources Industrialization, Nanjing 210023, China; 3 National and Local Collaborative Engineering Center of Chinese Medicinal Resources Industrialization and Formulae Innovative Medicine, Nanjing 210023, China

**Keywords:** licorice, salt stress, bioactive constituents, UFLC/MS/MS

## Abstract

The demand for licorice and its natural product derivatives in domestic and foreign market is considerably huge. The core production areas of licorice are covered with salinity and drought land in northwestern China. Studies have shown that suitable environmental stress can promote the accumulation of glycyrrhizin and liquiritin to improve its quality as medicinal materials. However, there are few reports on other bioactive constituents of licorice, not to mention their dynamic accumulation under stressed conditions. To explore the quality formation of licorice from the perspective of salt influence, a reliable method based on ultra-fast liquid chromatography tandem triple quadrupole mass spectrometry (UFLC–MS/MS) was established for simultaneous determination of sixteen bioactive constituents, including triterpenoids, flavonoids, chalcones and their glycosides. Physiological experiments were performed to investigate salt tolerance of licorice under different salinity treatments. The expressions of crucial genes (bAS and CHS), key enzymes of triterpenoid and flavonoid synthesis, were also tested by qRT-PCR. Our study found that 50 mM NaCl treatment (low stress) was the most favorable to promote the accumulation of bioactive constituents in the long term, without harming the plants. Flavonoid accumulation of non-stressed and low-stressed groups became different in the initial synthesis stage, and glycosyltransferases may have great influence on their downstream synthesis. Furthermore, bAS and CHS also showed higher levels in low-stressed licorice at harvest time. This work provides valuable information on dynamic variations in multiple bioactive constituents in licorice treated by salt and insight into its quality formation under stressed conditions.

## 1. Introduction

Licorice is one of the most frequently used herbs across the world. It was originally recorded in Shennong Materia Medica in China [1]. According to traditional Chinese medical theory, licorice can enhance “qi”(a kind of energy), relieve pain, act as an effective antidote [2,3], and it has also been widely applied to treat respiratory infections, gastritis, tremors, and peptic ulcers in folk medicine [4]. At present, people employ ingredients extracted from licorice as food additives, such as a flavoring agent in baked goods, frozen dairy products, and beverages [5]. Although there are different ways of using licorice, it is directly or indirectly related to its phytochemicals [6]. However, the quality of licorice on the market is uneven and evidence on its quality formation is scarce, seriously hindering the wide application of clinical and other aspects of licorice. Currently, more than 400 compounds are found in licorice. Triterpenoids and flavonoids take the largest proportion of all chemical compounds and contain the most diverse structure, along with their corresponding glycosides [7,8,9,10], which are dominantly responsible for various bioactivities. Therefore, the synthesis and accumulation of triterpenoids and flavonoids are an important aspect for the study of licorice quality formation.

Previous studies have shown that abiotic stress, such as salt or drought stress, significantly influences the contents of glycyrrhizin and several flavonoids in licorice [11,12], illustrating that the planting environment possibly leads to different accumulation of bioactive constituents of licorice, which results in differences in quality. Meanwhile, it is such environmental stress that helps the medicinal plants accumulate secondary metabolites, considering production areas of licorice, covered with salinity and drought land [13,14,15]. In addition, formation and accumulation of these metabolites is closely related to expression of key enzymes encoded by specific genes in biosynthetic pathways [11]. There are a great number of studies on the quality differences of licorice [16,17,18]. However, research on how the differences form under stress condition still remains limited. Accordingly, we investigated dynamic variations in multiple bioactive constituents, as well as the expression of rate-limiting enzymes associated with the synthesis of flavonoids and triterpenoids under salt stress. 

In the present study, sixteen bioactive constituents, including triterpenoids, flavonoids, chalcones and their glycosides, in licorice under salt stress were chosen to be determined by the valid ultra-fast liquid chromatography tandem triple quadrupole mass spectrometry (UFLC–QTRAP-MS/MS) method. Then we investigated the influence of NaCl stress on the bioactive constituents in licorice from total accumulative contents, the dynamic changes of bioactive constituent accumulation, and the different synthesis stage of bioactive constituents. Physiological characteristics of antioxidative enzymes such as peroxidase (POD), superoxide dismutase (SOD), glutathione reductase (GR), and catalase (CAT) activities were introduced. Changes of those enzymes were observed in licorice during different stress periods. At the gene level, the differential expressions of crucial genes (bAS and CHS), key enzymes of triterpenoid and flavonoid synthesis, were determined by qRT-PCR as well. 

Taken together, we studied dynamic changes of multiple bioactive constituents and their total accumulation in licorice under salt stress for the first time. Combined with the expression of rate-limiting enzymes encoding protein synthase of flavonoids and triterpenoids, our study not only provides valuable information on synthesis and accumulation of bioactive constituents in licorice, but insights of its quality formation research under stressed conditions. 

## 2. Results and Discussion

### 2.1. Effects of Salt Stress on Phenotype and Antioxidant Enzymes

**Figure 1:**
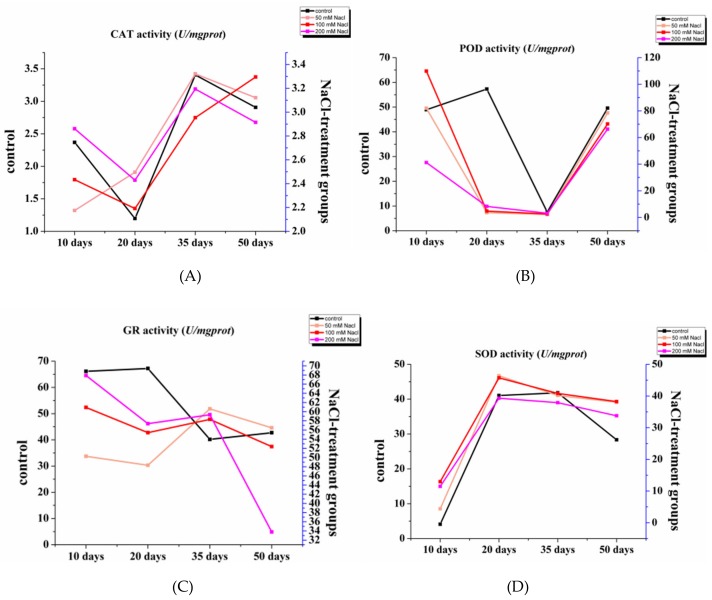
Dynamic contents of antioxidases CAT (**A**), GR (**B**), POD (**C**), and SOD (**D**) in all licorice groups of different NaCl concentrations.

The phenotype of the plants was obviously changed by salinity in the first place. In this study, morphological changes were observed in licorice seedlings under different concentrations of NaCl treatment. Both control (0 mM NaCl) and severe stress (200 mM NaCl) groups of seedlings showed weaker development than the other two stressed groups (Figure S1), suggesting licorice may live more vigorously under suitable salt conditions. Apart from growth inhibition, salinity stress also causes ion toxicity at the cellular level [19,20]. In order to survive harsh circumstances, plants have developed an efficient defensive strategy to scavenge ROS by producing a series of enzymes such as CAT, SOD, POD, etc. [21]. Besides, evidence indicates that the GR enzyme plays a crucial role in balancing the reduced glutathione/oxidized glutathione (GSH/GSSG) ratio, which participates in ROS scavenging by functioning as a redox buffer under NaCl stress conditions [22]. Figure 1 shows that the contents of those active enzymes fluctuated in different stressed periods. The POD and GR contents in the control group showed opposite trends to the stress group, indicating that antioxidants were activated in the salt-stressed groups, and their anti-stress activity showed a dynamic change. Furthermore, with the extension of stress time (starting from 35 days), the activity of enzymes in the low salt-stressed group (50 mM NaCl) basically showed slightly higher contents than those of other groups, meaning that salt stress in suitable concentrations had a lasting effect on plants. 

### 2.2. Dynamic Variations in Multiple Bioactive Constituents

#### 2.2.1. Optimization of UFLC–QTRAP-MS/MS Conditions

**Figure 2:**
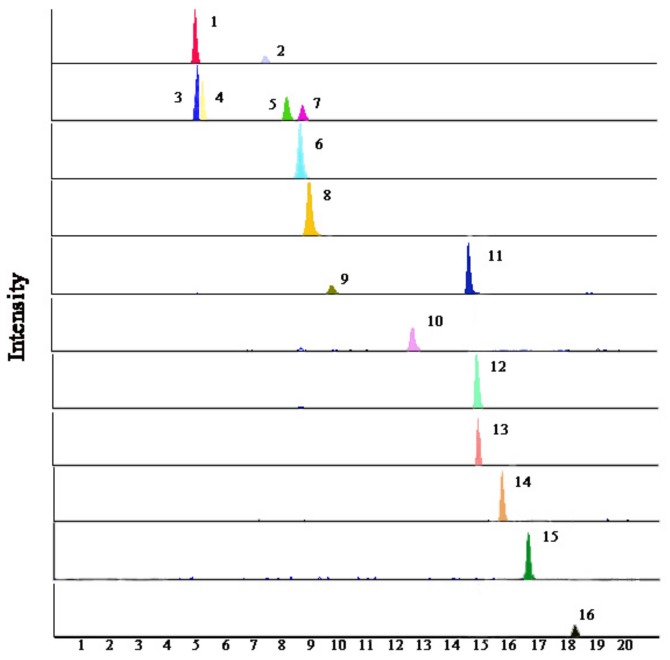
Multiple reaction monitoring (MRM) chromatogram of the sixteen compounds investigated in licorice.

The optimum chromatographic condition was modified according to our previous work [23]. The UFLC system with a XBridge^®^ C_18_ column (100 × 4.6 mm, 3.5 µm) was applied. Based on sensitivity and separation, satisfactory UFLC conditions were obtained when eluted with water containing 0.1% (v/v) formic acid and acetonitrile at 0.8 mL min^−1^ with a column temperature of 30 °C. MS condition was also studied to obtain the best instrumental conditions. After trial and error inspection, all of the detected chemicals (about 100 ng/mL) were injected separately into the electrospray ionization (ESI), and they all showed good condition in the negative ion mode. MRM (multiple reaction monitoring) transition from MS/MS was selected when the most abundant, specific, and stable fragment ions appeared. All of the optimum values for each compound are summarized in Table S1 and the representative extract ion chromatograms of the sixteen analytes with MRM mode are shown in Figure 2. 

#### 2.2.2. Method Validation

Method validation for UFLC/MS/MS analysis was carried out in aspects of linearity, sensitivity (LOD and LOQ), intra- and inter-day precision, repeatability, stability, and recovery test. All analytes showed satisfactory linearity with r ≥ 0.9990 within wide test ranges. The LODs and LOQs for all analytes ranged from 0.14–2.03 ng/mL^−1^ and 0.46–6.77 ng/mL^−1^ of the sixteen analytes, respectively, suggesting high sensitivity of the method. The intra- and inter-day precisions, repeatability, and stability were presented along with RSDs values less than 3.12%, 4.41%, 3.84%, and 4.17%, respectively. The mean recoveries of all analytes lay between 97.13% and 106.03% with a result of RSD values less than 4.35%. The results indicate that the developed method was qualified for quantitative analysis of the targeted compounds. The detailed results are shown in Table 1.

#### 2.2.3. Dynamic Accumulation in Bioactive Constituents

**Figure 3:**
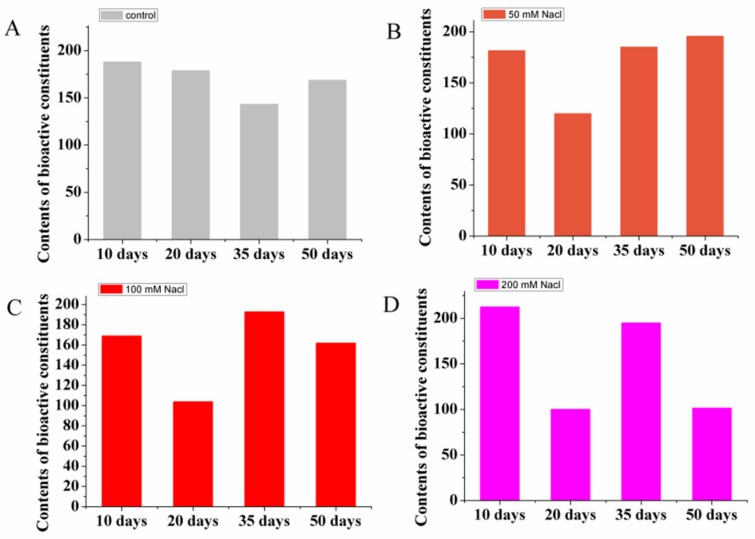
Dynamic accumulation of the total contents of the sixteen compounds. Control group (**A**). Salt-stressed groups: (**B**) 50 mM NaCl; (**C**) 100 mM NaCl; (**D**) 200 mM NaCl.

The validated UFLC–QTRAP-MS/MS method was then employed to comprehensively evaluate bioactive constituents in licorice under different salt treatments. Contents of the sixteen constituents, including triterpenoid saponins and flavonoids, were analyzed. These compounds have been demonstrated to contribute to pharmacological activities and quality evaluation in licorice [24,25]. Figure 3 shows that total amount of bioactive constituents in all groups fluctuated, and accumulation patterns of these constituents in different groups were diverse. The trends of the moderate and severe salt-stressed groups were similar, while the control group suggested opposite results during the later stages. The low salt group showed an overall upward trend, and these results indicate that the environment exerted impact on the dynamic accumulation of bioactive constituents and licorice of the four groups of NaCl treatments showed different accumulation patterns. Under high salt stress, the accumulation of these constituents in licorice displayed the most fluctuated variations, resulting in damage to the plant homeostasis and unstable accumulation of bioactive constituents.

**Figure 4:**
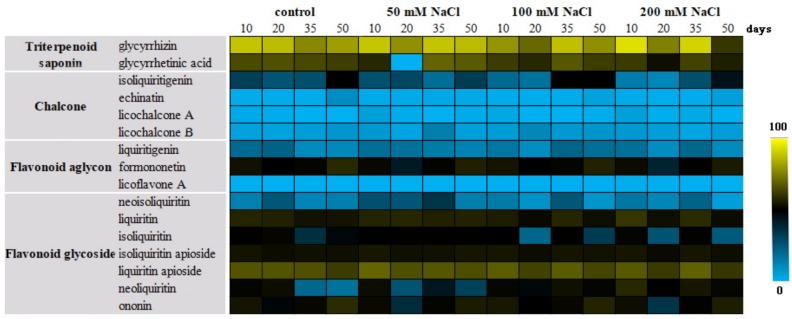
Heat map of the sixteen compounds during 50 days in different groups.

Based on the different accumulation of bioactive constituents in all groups of licorice, detailed changes about them during 50 days’ treatment were also investigated. As shown in Figure 4, the content distribution of the sixteen compounds in each group was similar, suggesting that the environment had little effect on the ratio of these bioactive constituents in non-treated or treated licorice. Among them, the contents of triterpenoid saponins and flavonoid glycosides were the highest in each group. Specifically, the content of glycyrrhizin, glycyrrhetinic acid, and liquiritin apioside took the first three places, followed by flavonoid glycosides such as liquiritin, isoliquiritin apioside, and ononin. In addition, all of the flavonoid aglycones and chalcones stayed at a low level, except for formononetin.

#### 2.2.4. Dynamic Distribution Patterns of Bioactive Constituents

**Figure 5:**
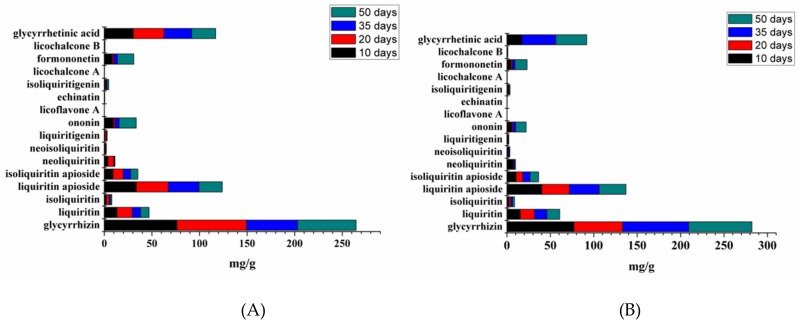

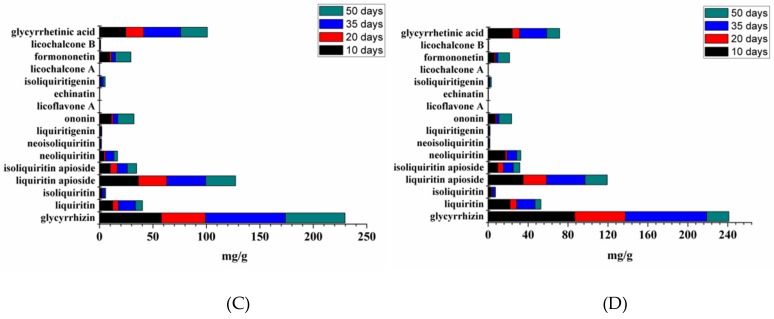
Accumulative contents of the sixteen compounds in different times: (**A**) control group; (**B**) 50 mM NaCl; (**C**) 100 mM NaCl; (**D**) 200 mM NaCl.

Overall, there is a difference in the total amount of the sixteen bioactive constituents among the stressed and non-stressed groups, indicating that different concentrations of NaCl significantly affected the accumulation of bioactive constituents in licorice (Figure 5). In terms of glycyrrhizin and glycyrrhetinic acid, the total accumulation of them was found with the highest content in the severe-salt and low-salt groups, and the middle-salt group showed the lowest level. In each group, the contents of bioactive constituents on the same synthesis pathway displayed a similar change trend, such as glycyrrhizin and glycyrrhetinic acid, liquiritin apioside and isoliquiritin apioside, and ononin and formononetin, which means that the accumulation of pathway-related constituents interacted with each other. Considering the results of the total contents of bioactive constituents, strengthening the degree of stress could be a wise strategy in the short term. However, in the long run, salinity treatment with 50 mM NaCl could better increase the accumulation of bioactive constituents without harming the plants.

#### 2.2.5. Comparison of Dynamic Accumulation in Flavonoids and Triterpenoids

**Figure 6:**
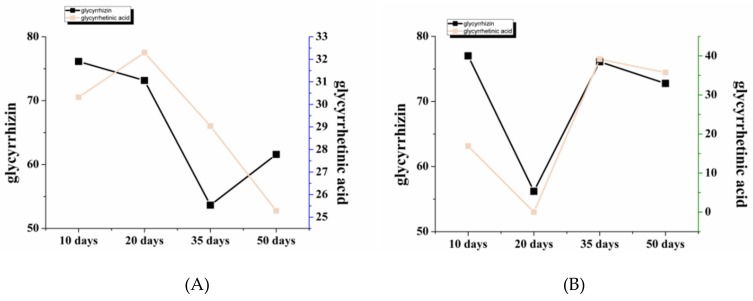

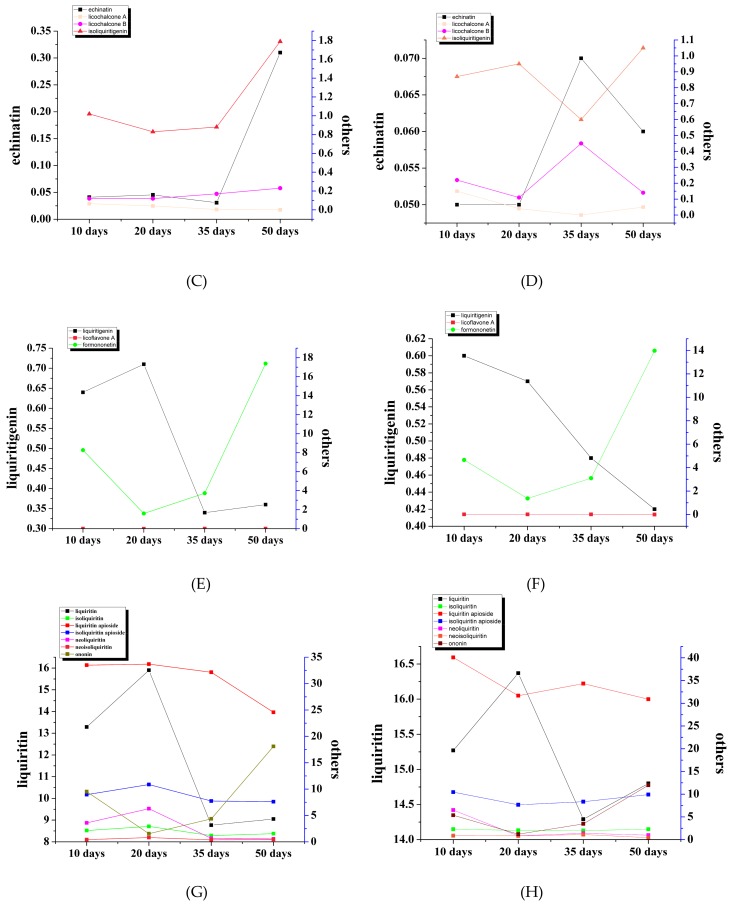
Dynamic changes of triterpenoid contents, chalcone contents, flavonoid aglycon contents and flavonoid glycoside contents in the control (**A**,**C**,**E**,**G**) and low-salt (**B**,**D**,**F**,**H**) groups.

According to the harvest period (i.e., after 50 days), plant growth status, and accumulation of bioactive constituents, the low-salt group was chosen for comparison with the non-stressed group for further study. Beginning with triterpenoid saponin, similar changes were observed in both groups, probably because glycyrrhetinic acid is a precursor of glycyrrhizin synthesis (Figure 6A,B) [26,27]. Specifically, the control group of triterpenoid saponin displayed an overall downward trend, and the low-salt group fluctuated, but eventually the low-salt group accumulated more of these two triterpenoids. Chalcone is the initial stage of flavonoid synthesis [28]. It can be seen form Figure 6C,D that the two groups have opposite trends, indicating that the accumulation of flavonoids became different in the initial synthesis. Flavonoid aglycones are represented by liquiritigenin, which is one of the key compounds in the synthesis of other flavonoids. At this stage, the content of liquiritigenin in both groups of licorices showed a falling trend (Figure 6E,F). Similar to chalcone, the total amount of the flavonoid aglycone in the control group was also slightly higher than that of the low-salt group. In the downstream of flavonoid synthesis, flavonoid glycosides are a class of constituents with high content, as well as more bioactivity studies in licorice. Their synthesis is involved in various glycosyltransferases [29,30], which are responsible for various kinds of synthesis of glycosides, including liquiritin and glycyrrhizin. These glycosyltransferases may play an important role in the late synthesis step. Figure 6G,H suggests that the changes in the content of flavonoid glycoside in the two groups are basically similar but not entirely the same. The total content of flavonoid glycosides in the stressed group was higher than those in the non-stressed licorice group, suggesting that the bioactive constituents ultimately showed different accumulation in the two groups probably due to synthetic pathways and the effects of the involved synthetase. 

### 2.3. Differential Expression Analysis of Key Enzymes in Flavonoid and Triterpenoid Biosynthesis

**Figure 7:**
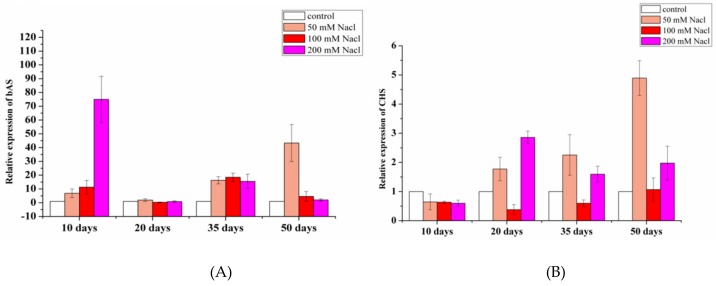
Dynamic changes of bAS and CHS expressions in control (**A**) and low-salt (**B**) groups.

RT-PCR was performed for two crucial genes (bAS and CHS), encoding rate-limiting enzymes of glycyrrhizin and flavonoid biosynthesis pathways [31,32]. Figure 7 illustrates that their expressions also have different fluctuation degrees after salt treatments. In the short term, the expressions of bAS and CHS were higher in the high salt-stressed group, and the low salt-stressed group was on the rise during the stressed period. The expressions of two genes in the moderate salt-stressed group were the worst, especially in the level of CHS, which was lower than the control group. As for bAS, its expression in all of the stressed groups displayed higher than those in the non-stressed group, and the total expression level was far greater than CHS, indicating that the saline environment significantly enhanced expression levels of these two key genes, particularly in respect to bAS. It is worth noting that the expression levels of bAS and CHS displayed highest in the low-stressed group at harvest time (after 50 days). Combined with the accumulation of bioactive constituents, the findings indicate that suitable salt stress could significantly affect the synthesis of flavonoids and glycyrrhizin.

## 3. Materials and Methods

### 3.1. Plant Materials and Salinity Treatments

The one-year-old licorice cultivars, botanically originated from *Glycyrrhiza uralensis* Fisch, were collected from Yanchi County, Ningxia Province, China. The plants of these licorice seedlings with close diameter and numbers of bud were selected for study. They were planted in the Medicinal Botanical Garden of Nanjing University of Traditional Chinese Medicine (latitude 118°57′1′′, east longitude 32°6′5′′) under a shelter of transparent film blocking off rainwater. Other conditions were almost equivalent to open air. Two seedlings of licorice were planted in one pot (height 50 cm, top diameter 30 cm, bottom diameter 25 cm) with approximately 25 kg dry soil. 

Experimental licorice were allowed to grow naturally until they were alive and germinated. Four levels of salt concentrations were then designed as follows: 0 mM (control group), 50 mM (low stress), 100 mM (moderate stress), and 200 mM (severe stress). NaCl treatments were conducted with 4 replicates at each concentration level. In order to avoid osmotic shock, the concentrations of salt increased gradually until the designated concentrations were reached. The whole period of stress lasted 50 days. Ultimately, experimental samples were harvested, cleaned with PBS, and quickly frozen with liquid nitrogen for subsequent experiments, including physiological assay, quantitative analysis, and the rest were collected as the voucher specimens. 

### 3.2. Physiological Experiment

Fresh leaves of licorice under different concentrations of salt treatment were weighed (approximately 0.5 g) and ground with liquid nitrogen. The powder was mixed with 4.5 mL of 0.1 mol/L PBS (PH7.4) and the mixture was centrifuged at 3500 rpm for 10 min. The supernatant was collected as the crude enzyme extract. SOD activity was measured according to the hydroxylamine method, POD activity was determined by the colorimetric method, and GR activity was determined by the method of Schaedle and Bassham. CAT activity was assayed in light with the ammonium molybdate method [19,33]. All of the experiments were carried out by assay kits obtained from Nanjing Jiancheng Bioengineering Institute (Nanjing, China), and 200 µL of each reaction solution was detected under UV-visible absorptions by a multi-mode microplate reader (SpectraMax M5, San Jose, CA, USA).

### 3.3. Multiple Bioactive Constituents Assay

#### 3.3.1. Chemicals and Reagents 

Reference compounds of the sixteen chemicals of liquiritin apioside (1) and isoliquiritin apioside (4) were purchased from Nanjing Jingzhu Bio-technology Co., Ltd. (Nanjing, PR China); neoliquiritin (2), ononin (6), licochalcone B (8), isoliquiritigenin (11), glycyrrhizin (12), formononetin (13), and licoflavone A (14) were acquired from Chengdu Chroma-Biotechnology Co., Ltd. (Chengdu, PR China); liquiritin (3), liquiritigenin (9), echinatin (10), licochalcone A (15), and glycyrrhetinic acid (16) were obtained from Liangwei Chemical Reagent Co., Ltd. (Nanjing, PR China); isoliquiritin (5) and neoisoliquiritin (7) were offered by Chengdu Purechem-Standard Co., Ltd. (Chengdu, PR China). The purities of all chemical standards were greater than 98% by HPLC analysis. Ultra-pure water was prepared by a Milli-Q purifying system (Millipore, Bedford, MA, USA). Methanol and acetonitrile of HPLC grade were purchased from Merck (Damstadt, Germany). All other chemicals used in the experiments were of analytical reagent grade (Shanghai Yuanye-Biotechnology Co., Ltd., PR China).

#### 3.3.2. Sample and Standard Solutions Preparation

Samples of all groups were harvested and naturally dried. After passing through a 60-mesh sieve, approximately 0.5 g of each sample powder was accurately weighed and ultrasonically extracted in 25 mL 70% methanol for 1 h. After cooling down at room temperature, the mixture was supplemented with 70% methanol to compensate for the lost weight, and subsequently centrifuged at 12,000 rpm for 10 min. The supernatant was collected, diluted tenfold, filtered through 0.22 µm membrane (Jinteng laboratory equipment Co., Ltd., Tianjin, China), and stored at 4 °C prior to UFLC–QTRAP-MS/MS analysis. 

The sixteen reference compounds were prepared by completely dissolving in methanol, and their concentrations were as follows: (1) 1.060 mg/mL; (2) 1.175 mg/mL; (3) 5.290 mg/mL; (4) 5.130 mg/mL; (5) 1.088 mg/mL; (6) 5.220 mg/mL; (7) 1.190 mg/mL; (8) 1.160 mg/mL; (9) 1.930 mg/mL; (10) 1.120 mg/mL; (11) 0.9600 mg/mL; (12) 10.05 mg/mL; (13) 1.330 mg/mL; (14) 1.010 mg/mL; (15) 0.950 mg/mL; (16) 5.710 mg/mL. This solution was further diluted with methanol for the establishment of the calibration curves. All of the solutions were stored at 4 °C before analysis.

#### 3.3.3. Chromatographic and Mass Spectrometric Conditions

The mobile phase was composed of acetonitrile (A) and 0.1% aqueous formic acid (B, v/v) with a linear gradient program: 0–4 min: 2% B; 4–6 min: 2–20% B; 6–9 min: 20–25% B; 9–10 min: 25–35% B; 10–12 min: 35% B; 12–15 min: 35–98% B; 15–16 min: 98–2% B; 16–20 min: 2% B. The flow rate was kept at 0.8 mL min^−1^ and the column temperature was maintained at 35 °C. The injection volume of the sample was 2 µL.

A triple quadrupole-linear ion trap mass spectrometer (QTRAP 5500) (AB Sciex, Framingham, MA, USA), equipped with an electrospray ionization (ESI) source, operated in the negative ion mode. The MS parameters were set as follows: GSI flow, 65 L min^−1^; CUR flow, 30 L min^−1^; gas temperature, 550 °C; pressure of nebulizer of MS, 5500 V (positive) and−4500 V (negative). To ensure mass accuracy and reproducibility, all MS data were acquired by the Analyst 1.6.3 software. The cone voltage and collision energy parameter of each compound were individually optimized to ensure mass accuracy and reproducibility.

#### 3.3.4. Method Validation and Sample Determination

The developed analysis method was validated for the linearity by the construction of calibration curves of each analyte. The limit of detection (LOD) and limit of quantitation (LOQ) were determined using a series of diluted standard solutions until the signal-to-noise (S/N) ratio was about 10 and 3, respectively. To evaluate accuracy, a recovery test was conducted by standard protocol and calculated by the formula: Recovery (%) = (found amount – original amount)/spiked amount × 100% [17]. To confirm the repeatability, six independent sample solutions from the same batch were processed in parallel and analyzed. As for precision, six replicate injections of mixed standard solution were analyzed within one day, and nine replicates on three consecutive days. Stability was achieved by analyzing the same sample solution, stored at room temperature at 0, 2, 4, 8, 12, and 24 h [34]. Relative standard deviations (RSD%) were taken as measurements for all of the variations. The quantitative determination of the bioactive constituents of licorice was performed under the optimal condition by QTRAP 5500.

### 3.4. Quantitative Real-Time PCR

We selected two genes (bAS and CHS) for quantitative real-time PCR (qRT-PCR) analysis, whose counterpart proteins are key enzymes in the biosynthesis of glycyrrhizin and flavonoid. The qRT-PCR reactions were carried out with a Trizol Total RNA Isolation Kit (Sangon Biotech, Shanghai, China) according to the protocols. cDNA pools for qRT-PCR from the total RNA were synthesized using HiScript Q RT SuperMix for qPCR (Vazyme Biotech Co.,Ltd, Nanjing, China). The primers were designed using Primer 3.0 softwaree (http://bioinfo.ut.ee/primer3-0.4.0/). The reference gene used β-actin. The relative expression level of genes was calculated using a real-time PCR system (ABI 7500 Real-Time PCR System) based on the 2^-ΔΔCt^ method. Primers for RT-qPCR are listed in Table S2.

### 3.5. Data Processing

UFLC/MS/MS data were acquired and analyzed by the Analyst 1.6.2 software. The bars, lines, and charts were charted by Origin pro 8 (Origin Lab, Massachusetts, USA). The mean values and standard deviation of all parameters were calculated by the measurements of four replicates.

## 4. Conclusions

Our study investigated the effects of salt stress on the bioactive constituents in licorice from three perspectives, including total accumulative contents, the dynamic changes of accumulation, and the different synthesis stages of bioactive constituents. The results showed that the categories and content ratio of bioactive constituents in each group were similar, meaning that the environmental impact did not dominate the distribution patterns of bioactive constituents in licorice, but the total amount of them in each group was different, suggesting salt stress indeed affected their accumulation. Specifically, more of glycyrrhetinic acid and glycyrrhizin were found in the salt stress groups, even if these two triterpenoids showed similar trends in both stressed and non-stressed licorice. The accumulation of flavonoids became different between stressed and non-stressed groups at the beginning of synthesis, and glycosyltransferases probably had great contribution to the accumulation of flavonoid glycosides, as well as glycyrrhizin. Furthermore, the low salt-stressed licorice performed best in expression levels of bAS and CHS. It also accumulated more bioactive constituents at harvest time. Severe stressed conditions can rapidly stimulate the increase of bioactive constituents and gene levels in a short stressed period, but it is not conducive to the growth of licorice. Thus, we believe 50 mM NaCl would be the best choice to breed licorice in the long run. Our work investigated dynamic variations in multiple bioactive constituents under salt stress and provided novel clues for the quality formation of licorice.

## Figures and Tables

**Table 1:** Regression equations, LODs, LOQs, intra- and inter-day precisions, repeatability, stability, and recovery for the 16 compounds.

No	Name	CAS No	Formula	Regression Equation	r	Linear Range (ng/mL)	LOD (ng/mL)	LOQ (ng/mL)	Precision		Repeatability	Stability	Recovery	
									Intra-day (RSD%; *n* = 9)	Inter-day (RSD%; *n* = 3)	(RSD%; *n* = 6)	(RSD%; *n* = 6)	%	RSD%
1	liquiritin apioside	74639-14-8	C_26_H_30_O_13_	Y = 115X + 127000	0.9997	97.7–200,000	0.32	1.08	2.43	3.93	2.74	3.93	101.38	3.12
2	neoliquiritin	5088-75-5	C_21_H_22_O_9_	Y = 245X + 2770	0.9999	122–250,000	2.03	6.77	2.77	3.62	3.30	2.14	101.18	2.42
3	liquiritin	551-15-5	C_21_H_22_O_9_	Y = 359X + 8000	1.0000	122–250,000	0.14	0.47	1.56	2.30	2.16	3.53	99.24	2.23
4	isoliquiritin apioside	120926-46-7	C_26_H_30_O_13_	Y = 173X − 114000	0.9997	97.7–200,000	0.55	1.85	2.48	2.53	3.32	1.39	98.07	3.04
5	isoliquiritin	5041-81-6	C_21_H_22_O_9_	Y = 605X − 51800	0.9997	97.7–100,000	0.22	0.74	2.70	4.41	2.55	2.80	99.13	2.39
6	ononin	486-62-4	C_22_H_22_O_9_	Y = 329X + 331000	0.9998	29.3–60,000	0.14	0.46	2.03	2.76	3.67	2.19	105.00	0.35
7	neoisoliquiritin	59122-93-9	C_21_H_22_O_9_	Y = 708X − 13700	0.9999	19.5–10,000	0.23	0.77	2.64	3.27	3.84	3.66	101.28	2.92
8	licochalcone B	58749-23-8	C_16_H_14_O_5_	Y = 315X − 8060	0.9996	1.5–3000	0.14	0.46	2.62	3.42	2.79	2.30	98.03	3.14
9	liquiritigenin	578-86-9	C_15_H_12_O_4_	Y = 688X − 73500	0.9995	156.2–20,000	0.25	0.82	2.85	2.53	1.84	4.17	97.41	2.02
10	echinatin	34221-41-5	C_16_H_14_O_4_	Y = 387X + 275	0.9997	0.5–1000	0.14	0.46	2.94	3.50	2.06	3.29	97.58	3.21
11	isoliquiritigenin	961-29-5	C_15_H_12_O_4_	Y = 1070X + 250000	0.9990	29.3–60,000	0.16	0.52	2.85	3.37	1.72	3.54	102.86	3.51
12	glycyrrhizin	1405-86-3	C_42_H_62_O_16_	Y = 51.9X + 913000	0.9990	1953–400,000	0.15	0.50	2.39	3.17	1.22	4.16	98.25	2.72
13	formononetin	485-72-3	C_16_H_12_O_4_	Y = 95X + 20900	1.0000	29.3–60,000	0.18	0.62	2.95	3.49	2.67	2.94	97.13	4.35
14	Licoflavone A	61153-77-3	C_20_H_18_O_4_	Y = 863X – 179	0.9991	1.56–200	0.13	0.43	2.80	2.95	2.79	2.15	96.83	2.61
15	Licochalcone A	58749-22-7	C_21_H_22_O_4_	Y = 561X − 556	0.9992	1.56–10,000	0.15	0.51	3.05	2.23	2.98	3.42	98.15	3.65
16	glycyrrhetinic acid	471-53-4	C_30_H_46_O_4_	Y = 0.0048X + 67.9	0.9994	146–300,000	1.40	4.68	3.12	3.13	2.88	2.72	106.03	3.26

## Supplementary Materials

The Supplementary Materials are available online.
