# Supplementary material for: Dynamic Variations in Multiple Bioactive Constituents under Salt Stress Provide Insight into Quality Formation of Licorice
Source: Molecules. 2019 Oct 11;24(20):3670. doi: 10.3390/molecules24203670 (PMC6832532; doi:10.3390/molecules24203670)
Supplement: Supplementary file 1 [file molecules-24-03670-s001.pdf]

Table S1. Optimized mass spectrometric parameters of sixteen metabolites

No	Name	CAS No	Formula	M.W.	MRM transitions(precursor → product)	tR (min)	DP/V	CE/eV
1	liquiritin apioside	74639-14-8	$C_{26}H_{30}O_{13}$	550.51	549.13/254.99	5.00	-70	-44
2	neoliquiritin	5088-75-5	$C_{21}H_{22}O_9$	418.39	417.09/255.10	5.05	-150	-20
3	liquiritin	551-15-5	$C_{21}H_{22}O_9$	418.39	417.08/255.06	5.23	-125	-26
4	isoliquiritin apioside	120926-46-7	$C_{26}H_{30}O_{13}$	550.51	549.098/255.01	7.42	-200	-38
5	isoliquiritin	5041-81-6	$C_{21}H_{22}O_9$	418.39	417.08/255.00	8.16	-165	-28
6	ononin	486-62-4	$C_{22}H_{22}O_9$	430.40	475.09/267.20	8.62	-30	-18
7	neoisoliquiritin	59122-93-9	$C_{21}H_{22}O_9$	418.39	417.09/254.90	8.71	-80	-22
8	licochalcone B	58749-23-8	$C_{16}H_{14}O_5$	286.28	285.05/149.90	8.93	-130	-30
9	liquiritigenin	578-86-9	$C_{15}H_{12}O_4$	256.25	254.99/135.00	9.73	-95	-20
10	echinatin	34221-41-5	$C_{16}H_{14}O_4$	270.28	269.14/91.99	12.54	-135	-36
11	isoliquiritigenin	961-29-5	$C_{15}H_{12}O_4$	256.25	255.09/119.00	14.48	-85	-32
12	glycyrrhizin	1405-86-3	$C_{42}H_{62}O_{16}$	822.93	821.35/351.00	14.78	-10	-56
13	formononetin	485-72-3	$C_{16}H_{12}O_4$	268.26	267.03/251.99	14.81	-140	-28
14	licoflavone A	61153-77-3	$C_{20}H_{18}O_4$	322.35	320.99/266.00	14.99	-125	-32
15	licochalcone A	58749-22-7	$C_{21}H_{22}O_4$	338.40	337.12/305.2	15.46	-45	-28
16	glycyrrhetinic acid	471-53-4	$C_{30}H_{46}O_4$	470.68	469.28/425.00	18.07	-210	-52

Note: M.W means the molecular weight; DP means declustering potential; CE means collision energy.
